# Unlocking the Potential of *Gracilaria chilensis* Against Prostate Cancer

**DOI:** 10.3390/plants14152352

**Published:** 2025-07-31

**Authors:** Verónica Torres-Estay, Lorena Azocar, Camila Schmidt, Macarena Aguilera-Olguín, Catalina Ramírez-Santelices, Emilia Flores-Faúndez, Paula Sotomayor, Nancy Solis, Daniel Cabrera, Loretto Contreras-Porcia, Francisca C. Bronfman, Alejandro S. Godoy

**Affiliations:** 1 Escuela de Química y Farmacia, Facultad de Ciencias, Universidad San Sebastián, Santiago 7510157, Chile; veronica.torres@uss.cl; 2 Centro de Biología Celular y Biomedicina (CEBICEM), Facultad de Ciencias, Universidad San Sebastián, Santiago 8580704, Chile; lorena.azocar@uss.cl (L.A.); maguilerao1@correo.uss.cl (M.A.-O.); cramirezs8@correo.uss.cl (C.R.-S.); lfloresf@correo.uss.cl (E.F.-F.); 3 Facultad de Ciencias Biológicas, Pontificia Universidad Católica de Chile, Santiago 8331150, Chile; camisch6@gmail.com; 4 Centro de Prevención y Control de Cáncer (CECAN), Pontificia Universidad Católica de Chile, Santiago 7810000, Chile; paulacsf@yahoo.com; 5 Departamento de Gastroenterología, Facultad de Medicina, Pontificia Universidad Católica de Chile, Santiago 8331150, Chile; nsolisl@gmail.com; 6 Centro de Investigación e Innovación Biomédica (CiiB), Universidad de los Andes, Santiago 7550000, Chile; dacabrera@uandes.cl; 7 Facultad de Ciencias de la Salud, Universidad Bernardo O’Higgins, Santiago 8370993, Chile; 8 Centro de Investigación Marina Quintay (CIMARQ), Facultad de Ciencias de la Vida, Universidad Andres Bello, Santiago 8370251, Chile; lorettocontreras@unab.cl; 9 Instituto Milenio en Socio-Ecología Costera (SECOS), Santiago 8370251, Chile; 10 Center of Applied Ecology and Sustainability (CAPES), Santiago 8331150, Chile; 11 Instituto de Ciencias Biomédicas, Facultad de Medicina, Universidad Andrés Bello, Santiago 8370186, Chile; francisca.bronfman@unab.cl

**Keywords:** cancer, prostate cancer, oleoresin, Gracilex^®^, seaweed

## Abstract

Prostate cancer (PCa) is the second leading cause of cancer-related death among men in most Western countries. Current therapies for PCa are limited, often ineffective, and associated with significant side effects. As a result, there is a growing interest in exploring new therapeutic agents, particularly from the polyphyletic group of algae, which offers a promising source of compounds with anticancer properties. Our research group has focused on investigating the effects of a novel oleoresin from *Gracilaria chilensis*, known as Gracilex^®^, as a potential therapeutic agent against PCa using both in vitro and in vivo models. Our findings indicate that Gracilex^®^ exhibits a time- and dose-dependent inhibitory effect on cell survival in LNCaP and PC-3 PCa, reducing viability by over 50% and inducing apoptosis, as evidenced by a significant increase in activated caspase-3 expression in both cell lines. Moreover, Gracilex^®^ significantly reduces the proliferation rate of both LNCaP and PC-3 prostate cancer cell lines, as evidenced by a marked decrease in the growth curve slope (*p* = 0.0034 for LNCaP; *p* < 0.0001 for PC-3) and a 40–50% reduction in the proportion of Ki-67-positive PCa cells. In addition, Gracilex^®^ significantly reduces in vitro cell migration and invasion in LNCaP and PC-3 cell lines. Lastly, Gracilex^®^ inhibits tumor growth in an in vivo xenograft model, an effect that correlates with the reduced PCa cell proliferation observed in tumor tissue sections. Collectively, our data strongly support the broad antitumoral effects of Gracilex^®^ on PCa cells in vitro and in vivo. These findings advance our understanding of its potential therapeutic role in PCa and highlight the relevance of further investigating algae-derived compounds for cancer treatment.

## 1. Introduction

Prostate cancer (PCa) is the second leading cause of cancer-related mortality in the United State [1,2]. According to Siegel et al. [2], projections for 2025 anticipate 313,780 new diagnoses and 35,770 deaths attributable to PCa. In Chile, data from the Global Cancer Observatory (Globocan) [3] for the year 2020 indicate that 8157 new cases were diagnosed, and 2296 individuals succumbed to PCa. Notably, prostate cancer ranks first in incidence and fourth in cancer-related mortality among men in Chile, with an adjusted mortality rate of 14.2 per 100,000 [3,4]. These statistics underscore the substantial impact of PCa on public health both in the United States and globally.

Currently, the serum prostate-specific antigen (PSA) diagnostic test plays a pivotal role in detecting the vast majority of PCa cases at an early stage, where the tumor is typically localized within the prostatic gland (referred to as organ-localized PCa) [5]. Standard treatments for organ-localized PCa involve aggressive treatments like radical prostatectomy and/or radiotherapy [6]. Currently, there are no approved chemotherapeutic or novel pharmacological treatments for early-stage PCa; although some immunotherapies are under investigation, they remain in experimental or clinical trial phases [7]. Despite these interventions, epidemiological studies reveal that between 30–50% of patients who undergo radical prostatectomy experience recurrence within 5–10 years, often manifested as metastatic or advanced disease [8]. For advanced or metastatic PCa, androgen deprivation therapy (ADT) remains the standard treatment. However, as a consequence of ADT, PCa progresses from an androgen-sensitive state to a more aggressive, and ultimately lethal, castration-resistant phenotype [9,10]. Current treatments, including ADT, often result in significant collateral morbidity, and regrettably, they generally fail to induce curative responses [11]. Therefore, there is a pressing need for more effective treatments that mitigate the limitations and side effects associated with existing therapeutic options [9,10,12].

As an alternative to conventional therapeutic approaches, especially for patients diagnosed with low and very low-risk PCa, which currently constitutes one-third of newly diagnosed PCa cases [13], active surveillance (AS) offers a continuous monitoring option [14,15,16]. This approach advocates for applying traditional treatments only in instances of disease progression [17,18,19]. Hence, these patients emerge as ideal candidates for a complementary treatment strategy, utilizing potential new compounds that are capable of impeding the advancement of the disease.

In recent years, cancer research has witnessed a notable shift towards investigating natural compounds sourced predominantly from plants, leading to the emergence of a category known as “phytopharmaceuticals” [20]. These compounds display a wide range of biological activities, encompassing antimutagenic, antioxidant, antiproliferative, anti-inflammatory, and/or antiangiogenic actions [21,22]. Moreover, they have the capacity to act at various stages during cancer progression [21]. Globally, diverse marine environments have proven to be rich sources of such compounds with therapeutic characteristics, as evidenced by discoveries in various species of algae [23,24]. These compounds demonstrate significant potential in combating various types of cancer, contributing to the ongoing exploration of innovative therapeutic approaches for PCa and other malignancies [25].

Seaweeds, also referred to as marine algae, have garnered significant attention in recent years due to their abundance of proteins, vitamins, minerals, and a diverse array of bioactive molecules in both hydrophilic and hydrophobic (oily) extracts [26,27,28]. Seaweeds are broadly categorized into three major groups based on their pigmentation, as follows: green (*Chlorophyta*), brown (*Phaeophyceae*), and red (*Rhodophyta*) algae [21]. Within the realm of oncology, particular attention has been focused on the utilization of extracts from red algae species, such as *Corallina officinalis* and *Palisada perforata* (formerly *Laurencia papillosa*). In the case of MCF-7 breast cancer cells, these extracts have demonstrated a detrimental impact on malignant breast cells by increasing their rate of apoptosis-mediated cell death [29,30]. Exploring the potential of brown algae, fucoxanthin, a carotenoid pigment, and its metabolites have demonstrated potent growth-suppressing properties in LNCaP and PC-3 PCa cell lines through the induction of G1-phase cell cycle arrest [31,32]. Additionally, 14-keto-stypodiol diacetate, a derivative from the alga *Stypopodium flabelliforme*, has been identified as a potent disruptor of microtubule cell organization. Consequently, it serves as an inhibitor of proliferation in DU-145 PCa cells [33]. Collectively, this body of evidence strongly supports the hypothesis that natural products derived from marine algae harbor significant anticancer activity. These effects are likely mediated through the activation of multiple mechanisms of antitumor action.

The edible Chilean red macroalga “*Gracilaria chilensis*”, commonly known as “pelillo”, has a rich history of use as both a food source and medicinal herb in Chile, dating back to pre-Hispanic times [34,35]. *Gracilaria chilensis* holds economic significance in the South Pacific region [36,37]. In addition to its nutritional potential, it is utilized for extracting agar hydrocolloids, contributing to the production of cosmetic products, the food industry, and even biomedical applications [35,38]. Our research group has identified and characterized an oily extract derived from *Gracilaria chilensis*, referred to as Gracilex^®^ [35]. This extract has been found to serve as a source of natural PPARγ ligands and antioxidants, showcasing potential benefits in mitigating metabolic disorders, while demonstrating a robust antioxidant activity [35].

In our current study, we focused on characterizing the antitumor properties of Gracilex^®^ using in vitro and in vivo models of PCa. Our results may contribute valuable insights into the potential therapeutic applications of Gracilex^®^ in the context of PCa treatment. These results are protected under the patent application number WO/2020/257952.

## 2. Results

### 2.1. Gracilex^®^ Reduces PCa Cell Survival by Inducing Apoptosis

**Figure 1:**
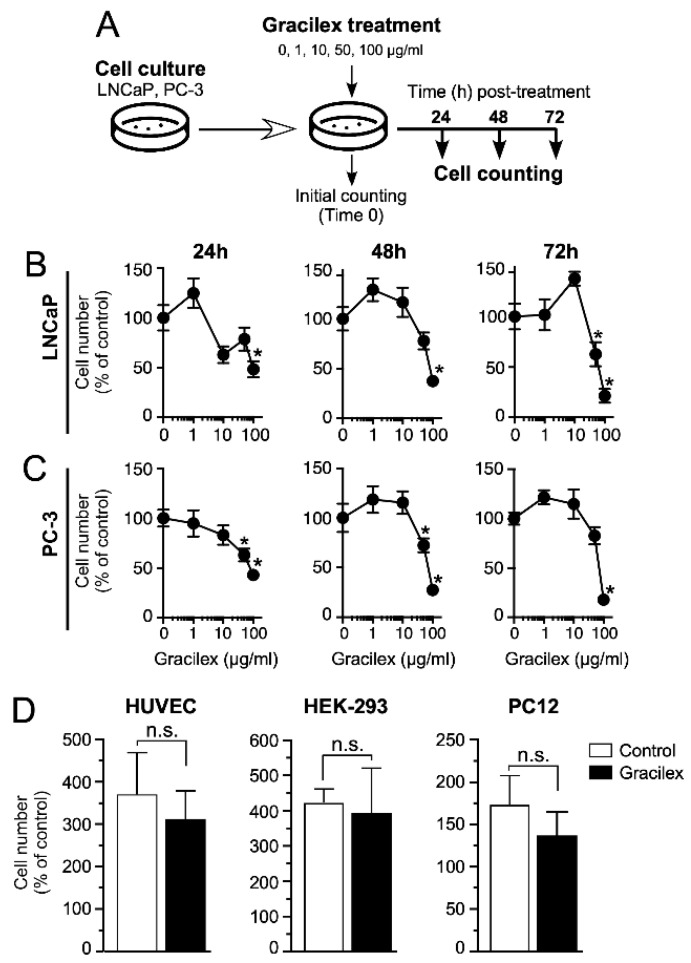
Impact of Gracilex^®^ on PCa cell viability. (**A**) Outline of the experimental procedure for treating and counting cells at 24, 48, and 72 h post-incubation with increasing concentrations of Gracilex^®^ (0, 1, 10, 50, and 100 µg/mL). The 0 µg/mL concentration corresponded to the vehicle control condition (0.01% DMSO). (**B**,**C**) Cell counts for LNCaP and PC-3 cells at 24, 48, and 72 h following Gracilex^®^ treatment. Data points represent the average of two independent experiments, each performed in triplicate. (**D**) Effect of Gracilex^®^ on non-malignant cells and non-prostatic tumorigenic cell lines. HUVEC, HEK293, and PC12 cells were treated with Gracilex^®^ (60 µg/mL), and cell counts were assessed at 48 h. Initial cell counts on day 0 were set as 100%. Statistical analysis was performed using a one-way ANOVA Test and a paired *t*-test. * *p* < 0.05. n.s.: not statistically significant.

To evaluate the effect of Gracilex^®^ on PCa cell viability, we performed an in vitro analysis using two human PCa cell lines with differing levels of aggressiveness, as follows: LNCaP (low-aggressiveness) and PC-3 (high-aggressiveness). Although both cell lines were originally derived from metastatic PCa lesions, they represent distinct disease stages. LNCaP cells, derived from a lymph node metastasis of a prostate adenocarcinoma, retain androgen sensitivity and are widely regarded as a model for early-stage, androgen-dependent disease with low metastatic potential. In contrast, PC-3 cells, established from a bone metastasis of an advanced prostate carcinoma, exhibit androgen independence, high metastatic potential, and aggressive behavior, making them a well-established model for advanced, castration-resistant prostate cancer [39]. The study included both a time-course evaluation at 24, 48, and 72 h, and a dose–response analysis using Gracilex^®^ concentrations of 1, 10, 50, and 100 µg/mL. The 0 µg/mL concentration corresponded to the vehicle control condition (DMSO) (Figure 1A). Our results demonstrated that Gracilex^®^ inhibited the survival of both PCa cell lines in a dose-dependent manner, with approximately 50% inhibition observed at 60 µg/mL (Figure 1B,C). This inhibitory effect was detectable within as early as 24 h and became more pronounced at 48 and 72 h post-treatment. Interestingly, at lower concentrations of Gracilex^®^ (1–10 µg/mL), we observed a slight, although not statistically significant, increase in the cell number in both LNCaP and PC-3 cells compared to the vehicle control condition. While this effect did not reach statistical significance, it may suggest a potential biphasic response to Gracilex^®^ treatment, which warrants further investigation. To evaluate the specificity of Gracilex^®^, we also assessed its effect on the survival of non-malignant human umbilical vein endothelial cells (HUVECs) and the following two additional non-prostatic tumorigenic cell lines: human embryonic kidney cells (HEK-293) and rat pheochromocytoma cells (PC12) (Figure 1D). Notably, Gracilex^®^ did not significantly affect the viability of HUVECs, HEK-293, or PC12 cells, suggesting a selective cytotoxic effect toward malignant PCa cells.

**Figure 2:**
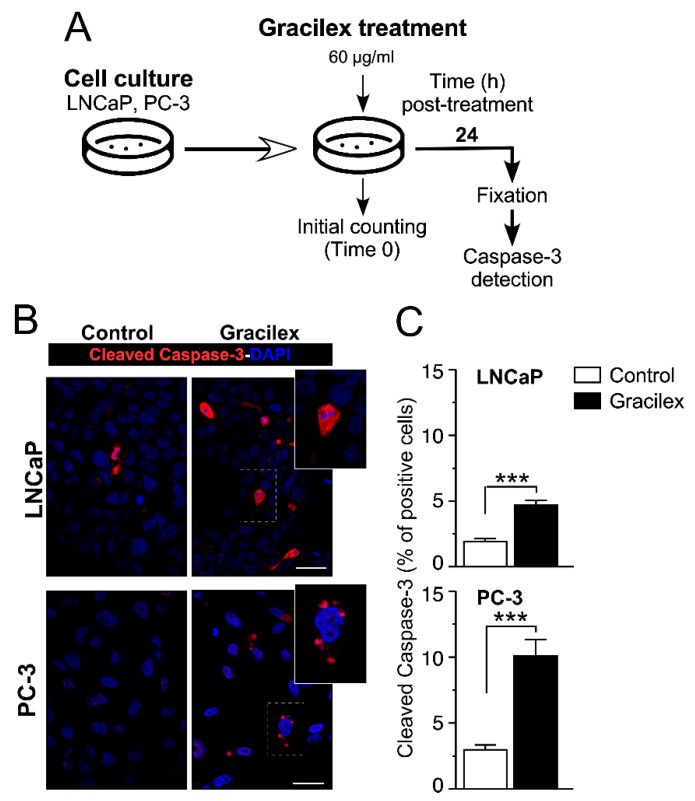
Gracilex^®^ induces apoptosis in PCa cells. (**A**) Schematic representation of the design for treating LNCaP and PC-3 cells with Gracilex^®^ (60 µg/mL) and assessing the expression of the apoptotic marker-activated caspase-3 after 24 h. (**B**) Immunofluorescence staining of activated caspase-3 in LNCaP and PC-3 cells, visualized using Alexa Fluor 594-conjugated anti-rabbit IgG. Nuclei were counterstained with DAPI. Scale bars: 20 µm. (**C**) Quantification of activated caspase-3-positive cells in LNCaP and PC-3 cell lines, expressed as a percentage of total cells based on DAPI staining. Statistical analysis was performed using an unpaired *t*-test. *** *p* < 0.001.

To confirm the impact on cell viability, we assessed the effect of Gracilex^®^ (60 µg/mL) on apoptosis in LNCaP and PC-3 cells by measuring the expression of the apoptotic marker-cleaved caspase-3 after 24 h of treatment (Figure 2A). Our results showed that Gracilex^®^ treatment significantly increased the expression of the apoptotic marker-cleaved caspase-3 compared to the control condition (Figure 2B). Quantification of the immunostaining results revealed that Gracilex^®^ elevated the percentage of apoptotic cells in both LNCaP and PC-3 cell lines cultured in vitro (Figure 2C). Human tonsil tissue sections were used as a positive control for the anti-cleaved caspase-3 antibody.

### 2.2. Gracilex^®^ Decreases Cell Proliferation in PCa Cells

**Figure 3:**
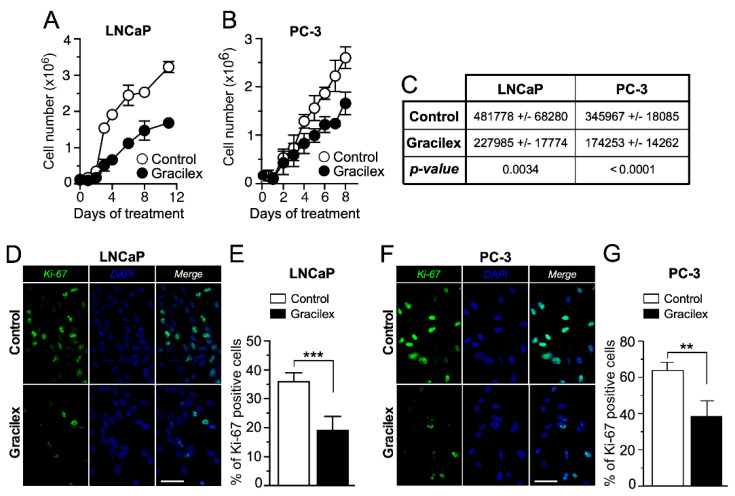
Effect of Gracilex^®^ on cell growth and proliferation in PCa cells. LNCaP (**A**) and PC-3 (**B**) cells were counted daily from day 0 to day 10 for vehicle (control) and Gracilex^®^-treated cells (60 µg/mL). Each data point represents the mean of three independent experiments performed in triplicate. (**C**) The table shows the changes in the slopes of each growth curve in the absence (control) and presence of Gracilex^®^ (60 µg/mL) for LNCaP and PC-3 cell lines. (**D**) Immunofluorescence analysis of the proliferation marker Ki-67 in LNCaP cells exposed to vehicle (control) or Gracilex^®^ (60 µg/mL). (**E**) Quantification of Ki-67-positive LNCaP cells, expressed as a percentage of the total cell population based on DAPI staining. (**F**) Immunofluorescence analysis of the proliferation marker Ki-67 in PC-3 cells exposed to vehicle (control) or Gracilex^®^ (60 µg/mL). (**G**) Quantification of Ki-67-positive PC-3 cells, expressed as a percentage of the total cell population based on DAPI staining. Nuclei were counterstained with DAPI. White scale bars: 20 µm. Statistical analysis was performed using an unpaired *t*-test. ** *p* < 0.005, *** *p* < 0.001.

To comprehensively characterize the effect of Gracilex^®^ (60 µg/mL) on the proliferation rate of PCa cells, we conducted cell growth curve analyses and immunostaining for the proliferation marker Ki-67 using the LNCaP and PC-3 cell lines (Figure 3A,B). Our results indicated that Gracilex^®^ significantly decreased the slope of the exponential phase of the growth curve for both LNCaP and PC-3 cells (Figure 3C), demonstrating a direct inhibitory effect on the proliferation rate of these PCa cell lines. In parallel, the effect of Gracilex^®^ on the expression of the proliferation marker Ki-67 in LNCaP and PC-3 cells demonstrated that the extract significantly decreased the percentage of Ki-67-positive cells in both the LNCaP (Figure 3D,E) and PC-3 (Figure 3F,G) cell lines. Additional studies assessing cell cycle and morphology were conducted on the LNCaP and PC-3 cell lines, yielding results consistent with those presented above. Together, these findings suggest that Gracilex^®^ exerts a measurable antiproliferative effect on PCa cells in vitro, which—alongside its pro-apoptotic activity—supports its potential as an anti-tumoral agent.

### 2.3. Gracilex^®^ Decreased PCa Cell Migration and Invasion Capacities

**Figure 4:**
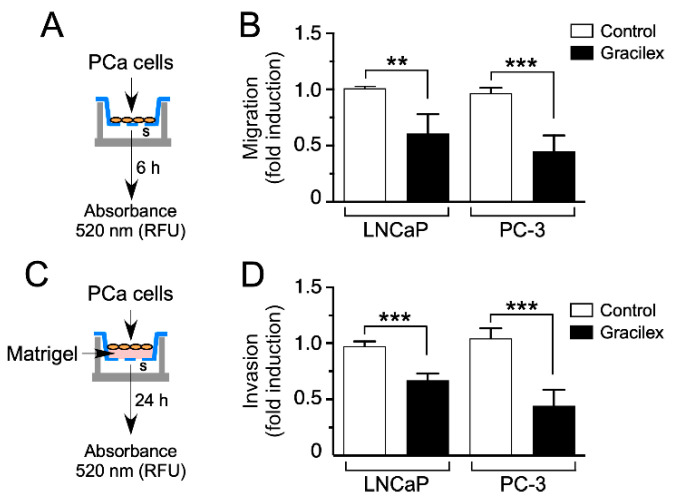
Effect of Gracilex^®^ on migration and invasion capacities in PCa cells. (**A**,**C**) Schematic representations of the experimental design for treating LNCaP and PC-3 cells with Gracilex^®^ (60 µg/mL) to assess their migration and invasion capacities. (**B**) Migration and (**D**) invasion capacities were measured using the CytoSelect™ Migration and Invasion Assay. Vehicle (control) or Gracilex^®^ treatments were applied to both the upper and lower chambers. FBS (serum, s) was used as a chemoattractant in the lower chamber. For invasion assays, the upper chamber contained a layer of Matrigel (**C**). The number of migrating/invading cells was quantified in the lower chamber using a fluorescent dye and measured with a Synergy Plate Reader. Statistical analysis was performed using an unpaired *t*-test. ** *p* < 0.005, *** *p* < 0.001.

To deepen our understanding of the impact of Gracilex^®^ on PCa cells, a meticulous analysis was undertaken to evaluate its influence on cellular migration and invasion capacities. Employing a comparative study, we contrasted the effects of Gracilex^®^ with those of a control vehicle, aiming to elucidate specific alterations in the migratory and invasive behaviors of PCa cells in response to the Gracilex^®^ extract. For these analyses, fetal bovine serum (FBS) served as a chemoattractant, as illustrated in schematic representations (Figure 4A,C). Intriguingly, our findings reveal that Gracilex^®^ exerted a significant suppressive effect on cell migration and invasion capacities in the LNCaP and PC-3 cell lines (Figure 4B,D) compared to the control vehicle. Notably, the inhibitory effects were more pronounced in the highly aggressive PC-3 cells compared to the less aggressive LNCaP cells. These observations underscore the potential therapeutic significance of Gracilex^®^ in mitigating the migratory and invasive properties of PCa cells, particularly in the context of more aggressive phenotypes, such as those exhibited by PC-3 cells.

### 2.4. Gracilex^®^ Decreased Tumor Growth on a Cell Line-Derived Xenograft Model of PCa

**Figure 5:**
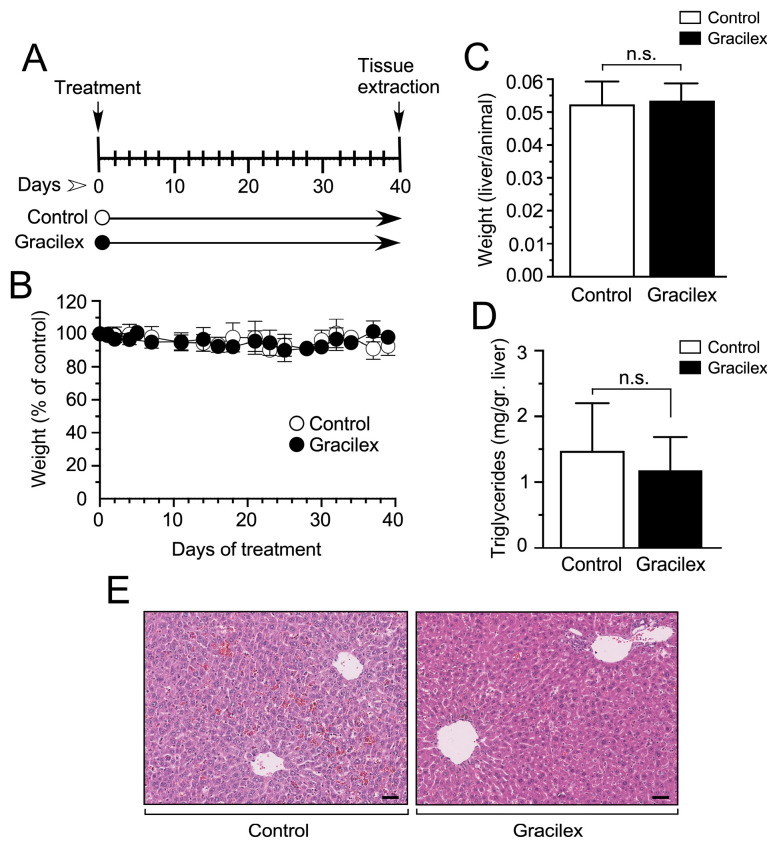
Effect of Gracilex^®^ on in vivo mouse models. (**A**) Schematic representation of the experimental design for treating mice with either vehicle (control) or Gracilex^®^ (300 mg/kg). (**B**) The effect of corn oil (control) or Gracilex^®^ treatment on the total body weight of mice, measured every 2 days for 40 days. (**C**) The liver-to-body weight ratio was measured at the end of the treatment period (40 days). (**D**) Triglyceride levels per gram of liver tissue were measured at the end of the treatment period (40 days). (**E**) Hematoxylin and eosin staining of liver sections from mice treated with vehicle (control) or Gracilex^®^. Scale bars: 40 µm. Statistical analysis was performed using an unpaired *t*-test. n.s. = not statistically significant.

**Figure 6:**
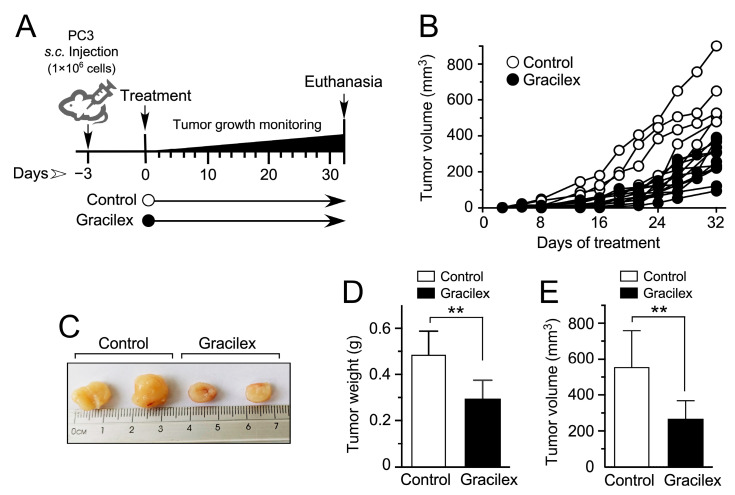
Effect of Gracilex^®^ on a cell line-derived xenograft model of human PCa. (**A**) Schematic representation of the experimental design for treating mice bearing PC-3 cell line-derived tumors with either corn oil (control) or Gracilex^®^ (300 mg/Kg) for 4–5 weeks. (**B**) Tumor growth of PC-3 cell line-derived xenografts was measured 2–4 times per week. (**C**) Representative images of dissected PC-3 cell line-derived tumors. (**D**,**E**) Tumor weight (g) and volume (mm^3^) were measured after dissecting tumors from mice treated with vehicle (control) or Gracilex^®^ (60 µg/mL). Statistical analysis was performed using an unpaired *t*-test. ** *p* < 0.005.

To provide insights into the effectiveness of Gracilex^®^ in a more physiologically representative setting, we conducted an in vivo study, assessing the impact of Gracilex^®^ on tumor growth/cell proliferation using an in vivo model involving the subcutaneous injection of PC-3 cells into the flank of six-week-old male NOD-scid IL2Rgammanull (NSG) mice (Jackson Laboratories). The administration of the Gracilex^®^ extract to the mice was conducted via gavage, with a frequency of three times per week for five weeks, employing a Monday–Wednesday–Friday schedule (15 doses) (Figure 5A and Figure 6A).

Initially, we assessed the systemic effects of Gracilex^®^ in these animals, as depicted in Figure 5. This comprehensive evaluation encompassed parameters such as weight loss, liver/animal weight ratio, liver tissue levels of triglycerides, and a detailed examination of liver tissue histology. The latter was meticulously analyzed by a skilled pathologist (DC) to ensure there was a thorough understanding of any potential impact on hepatic morphology. This systematic approach allows for a holistic assessment of Gracilex^®^ effects and provides crucial insights into its safety profile within the experimental model. Our findings revealed that Gracilex^®^ had no significant impact on animal weight throughout a 40-day treatment period. Animals treated with Gracilex^®^ exhibited comparable weights to the control group, which received vehicle (corn oil) via gavage (Figure 5A,B). This observation suggests a lack of systemic toxicity or adverse effects on weight regulation during the specified treatment duration. Furthermore, our investigation placed particular emphasis on assessing the impact of Gracilex^®^ on liver homeostasis. To achieve this, we conducted a detailed analysis, including evaluating the liver weight/animal weight ratio and a comprehensive examination of liver histology (Figure 5C,D). Our results indicated that either liver weight/animal weight ratio (Figure 5C) or liver tissue histology (Figure 5E) were not affected by Gracilex^®^. In the context of liver histology, the overall tissue architecture in the Gracilex^®^ condition remained intact compared to the control. This preservation is evident in both the portal spaces, the areas surrounding the central veins, and the sinusoidal spaces. No signs of hepatocellular injury or inflammatory infiltration were observed. The cytoplasm of hepatocytes exhibited uniformity without any abnormal accumulation of lipid droplets. The nuclei were well-defined, and there were no indications of cellular distress (Figure 5E). Finally, considering that our extract was oily, we specifically investigated the influence of Gracilex^®^ on liver tissue triglyceride levels, as illustrated in Figure 5D. Despite some degree of variability observed in the measurements under both control and Gracilex^®^ conditions (Figure 5D), our analysis revealed that Gracilex^®^ administration did not have a statistically significant effect on liver tissue triglyceride levels when compared to control conditions. Moreover, the evaluation of liver tissue histology indicated a minor impact to no impact on fat accumulation, providing additional evidence that Gracilex^®^ did not induce alterations in liver lipid content during the treatment period. These findings contribute to a comprehensive understanding of the metabolic impact of Gracilex^®^, emphasizing its potential safety profile concerning lipid metabolism and hepatic functions.

In the PC-3 cell line-derived xenograft model, the tumor volume was monitored three times a week over 5 weeks, as depicted in Figure 6A. Notably, approximately two weeks post-cell injection, discernible distinctions in the rate of tumor growth emerged between the Gracilex^®^ treatment group and the control condition, persisting throughout the entire experimental period (Figure 6B). After this observation period, tumors were dissected, measured, and weighed before undergoing fixation and inclusion for immunohistochemistry analyses. Noteworthy differences were evident when comparing tumors obtained from the Gracilex^®^ treatment condition to those from the control group. Tumors from the Gracilex^®^ condition exhibited a visually smaller size (Figure 6C), along with a significant decrease in both weight (Figure 6D) and volume (Figure 6E), compared to tumors from the control condition. This suggests that Gracilex^®^ may impact the growth characteristics of the PC-3 cell line-derived xenografts, resulting in a reduction in the size of the tumors.

**Figure 7:**
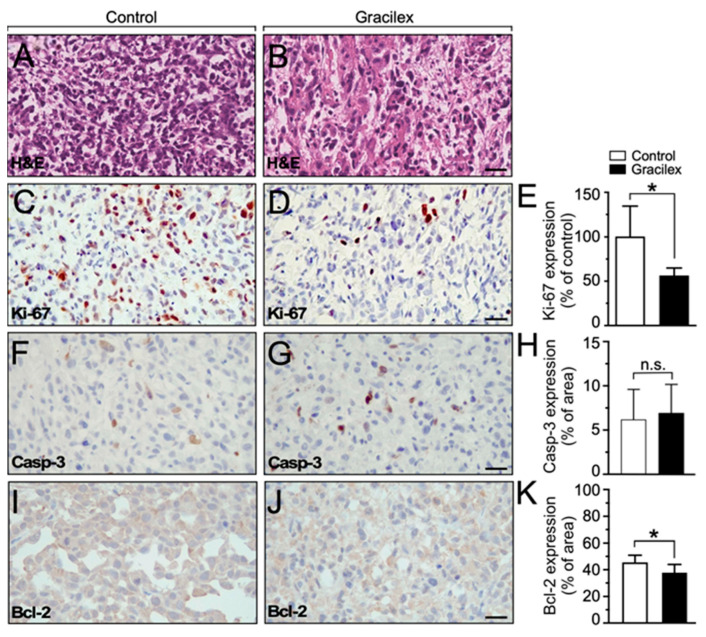
Effect of Gracilex^®^ on in vivo expression of cell proliferation and apoptotic markers. (**A**,**B**) Hematoxylin and eosin staining of tissue sections from cell line-derived xenograft tumors treated with either corn oil (control) or Gracilex^®^ (300 mg/Kg). (**C**,**D**) Immunohistochemical analysis of Ki-67 in tissue sections from cell line-derived xenograft tumors treated with either corn oil (control) or Gracilex^®^ (300 mg/Kg). (**E**) Quantification of Ki-67 immunostaining, expressed as the percentage of Ki-67-positive nuclei relative to the total cell population based on hematoxylin staining. (**F**,**G**) Immunohistochemical analysis of activated caspase-3 in tissue sections from cell line-derived xenograft tumors treated with either corn oil (control) or Gracilex^®^ (300 mg/Kg). (**H**) Quantification of cleaved caspase-3 immunostaining, expressed as the percentage of the total area of the tissue section. (**I**,**J**) Immunohistochemical analysis of Bcl-2 in tissue sections from cell line-derived xenograft tumors treated with either corn oil (control) or Gracilex^®^ (300 mg/Kg). (**K**) Quantification of Bcl-2 immunostaining, expressed as the percentage of the total area of the tissue section. Scale bars: 20 µm. Statistical analysis was performed using an unpaired *t*-test. * *p* < 0.05, n.s. = not statistically significant.

Histochemical analyses indicated that the xenograft tumors were predominantly composed of PCa epithelial cells under both control and Gracilex^®^ conditions, with no signs of excessive necrotic areas observed in either condition (Figure 7A,B). The subsequent immunohistochemistry analyses of the dissected tumors revealed that tumors originating from the Gracilex^®^ condition displayed a notably lower index of Ki-67 expression (Figure 7). Specifically, there was a reduction in the proportion of tumor epithelial cells in the Gracilex^®^ condition that exhibited Ki-67 immunostaining at their nuclei level (Figure 7C,E). This clearly contrasts with the control condition, where the Ki-67 expression was comparatively higher (Figure 7D,E). These findings align with our in vitro data, demonstrating that Gracilex^®^ treatment resulted in a decrease in the proliferation rate of PCa cells. Additionally, a subtle, although not significant, increase in activated caspase-3 immunostaining was observed in tissue sections from the Gracilex^®^ treatment compared to the control condition (Figure 7F–H). Conversely, a significant decrease in the anti-apoptotic protein Bcl-2 was noted in the Gracilex^®^ group relative to the control (Figure 7I–K). This convergence of in vitro and in vivo results reinforces the hypothesis that Gracilex^®^ mainly regulates the proliferation dynamics of PCa cells.

## 3. Discussion

Marine macroalgae have attracted unprecedented interest as health food and additives, predominantly because of their plethora of biological activities, including anti-cancer activities. Extracts from various seaweed species have shown cancer-fighting agents that inhibit key processes in cancer development and metastasis, such as cell proliferation, inflammation, and migration, as well as the induction of apoptosis [25,40,41,42]. Our study delves into the anticancer activities of an oleoresin from the macroalgae *Gracilaria chilensis* based on in vitro and in vivo experimental studies on PCa. Our findings demonstrate that Gracilex^®^ effectively reduces cell survival, proliferation, migration, and invasion in vitro and decreases tumor growth/cell proliferation in an in vivo xenograft model. These results suggest that Gracilex^®^ exerts a multi-faceted inhibitory effect on PCa, which may be beneficial in addressing different aspects of cancer progression. With less than 3% of the world’s marine macroalgal species assessed for anticancer activities [43], it is imperative that prospecting a more comprehensive marine species population be continually fostered.

Our in vitro analysis indicated that Gracilex^®^ decreases cell survival by inducing apoptotic cell death in both LNCaP (low aggressiveness) and PC-3 (high-aggressiveness) PCa cell lines. This was evidenced by the dose-dependent decrease in cell survival, as well as the increase in cleaved caspase-3 expression in LNCaP and PC-3 cells. The induction of apoptosis is one of the major chemopreventive mechanisms of natural products [44]. Notably, this apoptotic effect was observed at concentrations that did not significantly compromise the viability of non-malignant HUVEC cells, nor did it affect other non-prostatic tumorigenic cell lines, such as HEK-293 and PC12 cells. These findings highlight the selective cytotoxicity of Gracilex^®^ toward PCa cells, a highly desirable characteristic for a tumor-specific therapeutic agent. The ability of Gracilex^®^ to selectively target malignant prostate cells while sparing non-cancerous cells, such as HUVEC, underscores its potential as a safe anticancer agent. Nonetheless, we acknowledge that additional evaluations using non-tumorigenic prostatic epithelial cells are necessary to definitively confirm the tissue-specific selectivity of Gracilex®. This remains a key priority for future studies. This notion is further supported by the fact that Gracilex^®^ is derived from an edible macroalga. The anti-cancer activities observed with Gracilex^®^ are consistent with those obtained from extracts isolated from other members of red algae, such as porphyran and agar extracted from *Pyropia yezoensis* and *Kappaphycus striatum*. These extracts demonstrated inhibitory effects on the growth of cancer cells, including Hep3B, HepG2, MCF-7, K562, and HT-29 [45,46,47,48]. Moreover, porphyran was also non-toxic on normal cells and induced cancer cell death in a dose-dependent manner [47]. Interestingly, Gracilex^®^ did not affect the survival of other non-prostatic tumorigenic cell lines, suggesting a more prostate-specific mechanism of action. This specificity warrants further mechanistic investigation to elucidate the underlying pathways responsible for its selective cytotoxicity.

Our results also show that Gracilex^®^ significantly suppresses the proliferation of PCa cells, as demonstrated by the slowed cell growth and reduced Ki-67 expression in both LNCaP and PC-3 cell lines. The consistent reduction in proliferation observed across both low- and high-aggressiveness cell lines suggests that Gracilex^®^ may be effective in treating various stages of PCa. The observed decline in Ki-67 expression in xenograft tumors from Gracilex^®^-treated mice also aligns with our in vitro findings, corroborating the anti-proliferative effects of the extract in a physiologically relevant model. This is significant, since studies of seaweed extract for the treatment of PCa are scarce [31,45,48,49]. For example, there is evidence that extracts from a green alga (*Caulerpa lentillifera*) and compounds from brown algae (Fucoidan from *Undaria pinnatifida*) have shown activity against PCa, inducing apoptosis and effectively inhibiting the growth and colony formation of various PCa cell lines, with notable efficacy against androgen-sensitive LNCaP cells [45,49]. In contrast to these studies, our work represents a pioneering investigation into the lipidic fraction of red algae extracts and their effects on PCa. While the anticancer properties of red algae have been explored in previous research, mentioned above, these efforts have primarily focused on water-soluble fractions. To date, the specific evaluation of lipidic components from red algae in the context of PCa remains largely unexplored.

The present study demonstrates the ability of Gracilex^®^ to inhibit the migration and invasion of both LNCaP and PC-3 prostate cancer cells. Given the critical role of these processes in cancer metastasis, these findings provide preliminary support for the potential therapeutic use of Gracilex^®^ in preventing PCa dissemination. Although 60 µg/mL approximates the IC50 value, our data show that a viable population of cells remains under this concentration, allowing for the assessment of functional behaviors, such as migration. Several studies have demonstrated that seaweed extracts inhibit cancer cell migration and invasion; however, these findings have been limited only to in vitro models [25,41,50,51]. For example, Do Thi et al. [51] demonstrated that laver extract from the red alga *Pyropia tenera* inhibits the migration of SK-Hep1 cells in a wound migration assay and significantly reduces the number of invasive cells. The study further proved that the underlying mechanism involves inhibiting MMP-2 and MMP-9 activities. These metalloproteinases play critical roles in tumor proliferation, growth, and invasion [40,51].

The in vivo experiments using a PC-3 cell-derived xenograft model further confirmed the anti-tumor efficacy of Gracilex^®^. Oral administration of the extract significantly reduced tumor growth without inducing detectable systemic toxicity, as evidenced by the absence of significant changes in body weight, liver morphology, or circulating triglyceride levels. The lack of adverse effects on liver histology and lipid metabolism suggests that Gracilex^®^ does not elicit hepatic injury, a critical consideration in the development of safe cancer therapies. The observed reduction in tumor size and proliferative activity within the xenografts supports the notion that Gracilex^®^ primarily impairs cell division dynamics in PCa cells rather than inducing widespread cell death. Although increased apoptosis was detected by activated caspase-3 immunostaining, the effect was modest, further emphasizing the dominant contribution of anti-proliferative mechanisms to tumor inhibition. In previous studies, Gracilex^®^ demonstrated insulin-sensitizing and anti-inflammatory properties in high-fat diet-induced metabolic models [35]. The current findings expand its potential application to oncology, although differences in experimental context and target tissues make direct comparison challenging. Nonetheless, it is important to acknowledge the limitations of cell line-derived xenograft models, including their inability to fully recapitulate the complexity of the tumor microenvironment. Future studies employing more physiologically relevant systems, such as genetically engineered mouse models or patient-derived xenografts, will be instrumental in validating the therapeutic potential of Gracilex^®^ and enhancing the translational relevance of these findings.

Preliminary evidence on the chemical composition of Gracilex^®^ suggests that its components could have potential anti-cancer effects. For example, Gracilex^®^ contains high levels of gamma-tocopherol, one of the eight natural forms of vitamin E, a potent lipophilic antioxidant [35,49]. Various preclinical and cell-based studies have demonstrated that gamma-tocopherol may have chemosensitizing and anti-proliferative effects in PCa [52,53,54,55]. Interestingly, beta-carotene, the precursor of retinoic acid, is another component of Gracilex^®^ [35] and is metabolized in PCa cells, modulating gene expression [56]. Furthermore, Gracilex^®^ has been shown to contain natural activators of PPARgamma, a result that correlates with the presence of palmitic acid, oleic acid, and arachidonic acid, all-natural activators or precursors of natural ligands for PPARs [35,57]. PPARs are nuclear receptors regulating glucose and lipid metabolism, and PPARgamma is the molecular target of thiazolidinediones, drugs used for insulin and glucose sensitization [58]. Consistent with this, Gracilex^®^ acts as a glucose and insulin sensitizer in high-fat diet models in mice [35]. Various ligands of PPARgamma have been shown to have anti-cancer effects in cellular models of PCa [59,60], suggesting that there is a key link between lipid metabolism and PCa progression [61]. Interestingly, PPARs are nuclear receptors that heterodimerize with the retinoic acid receptor to exert different and broad actions in the metabolic states of PCa cells, including the regulation of glucose metabolism and the beta-oxidation of fatty acids [57]. Collectively, these findings suggest that the mixture of hydrophobic bioactive molecules present in Gracilex^®^, including potent antioxidants and natural PPARγ activators, may induce metabolic and transcriptional reprogramming in PCa cells, ultimately reducing their proliferative and pro-migratory phenotype. Further studies are warranted to dissect the specific contribution of individual Gracilex^®^ components to these biological effects and to elucidate the cellular mechanisms underlying its anti-proliferative properties. In addition, evaluating the efficacy of Gracilex^®^ in other PCa subtypes, including models of castration-resistant prostate cancer, would provide valuable insights into its broader therapeutic potential. Furthermore, considering the antioxidant nature of key Gracilex^®^ components, such as γ-tocopherol and β-carotene, it is plausible that modulation of oxidative stress may contribute to the observed pro-apoptotic effects. Future studies should investigate whether the redox-modulating properties of Gracilex^®^ play a direct role in activating apoptotic signaling pathways in prostate cancer cells.

In summary, our findings highlight the potent anti-tumor activity of Gracilex^®^ against PCa, primarily characterized by the induction of apoptosis, the suppression of cell proliferation, and the inhibition of migration and invasion. The extract’s selective cytotoxicity toward malignant cells, combined with the absence of significant toxicity in vivo, underscores its potential as a safe and effective therapeutic agent. Further mechanistic investigations and preclinical studies are warranted to advance Gracilex^®^ as a candidate for prostate cancer treatment.

### Limitations and Future Perspectives

Despite the promising results presented in this study, several limitations must be acknowledged. First, while the in vitro and in vivo data suggest that Gracilex^®^ exerts anti-tumoral effects on PCa models, the underlying molecular mechanisms remain to be fully elucidated. Additional studies focusing on the identification of specific molecular targets and signaling pathways modulated by Gracilex^®^ are warranted.

Second, although we observed significant effects in vivo, the dosage administered—300 mg/kg—represents a relatively high concentration when extrapolated to potential human application. It is important to emphasize that this dosage was selected based on the in vitro efficacy observed at concentrations of 60 µg/mL, as well as on doses previously reported in animal studies [35]. Nonetheless, we recognize that this represents a substantial dose for translational purposes, highlighting the need for future pharmacokinetic studies, dose–response analyses, and toxicity evaluations to establish an optimized and clinically relevant dosing regimen.

Finally, we acknowledge that our study focused primarily on evaluating the therapeutic potential of Gracilex^®^ in aggressive PCa models, and did not include direct comparisons with established chemotherapeutic agents or antiandrogenic therapies. Such comparisons would provide important context for assessing the relative efficacy of this extract and will be the focus of future research efforts. Collectively, these considerations outline critical next steps to further validate the potential of Gracilex^®^ as a therapeutic strategy for PCa.

## 4. Methods

### 4.1. Gracilex^®^ Extract Preparation

For in vitro studies, the light-sensitive Gracilex^®^ extract was collected and dried in dark glass vials. The dried extract was then reconstituted in 0.01% DMSO (CORNING) to a final concentration of 100 mg/mL. For in vivo administration, the extract was resuspended in corn oil (Sigma-Aldrich, St. Louis, MO, USA) at a ratio of 500 mg of extract to 638 mg of oil, as measured using an analytical balance [35]. The mixture was vortexed until homogeneous, the total volume was recorded, and the concentration of Gracilex^®^ per µL of oil was calculated. Stock solutions were sealed under nitrogen gas and incubated at 37 °C for 30 min in a temperature-controlled water bath. To prevent oxidative degradation, all preparations and manipulations were performed under minimal light exposure, and tubes were purged with nitrogen gas each time they were opened. The protocol for Gracilex^®^ oleoresin production is described in detail in patent WO/2014/186913 [35]. The chemical composition of Gracilex^®^ was previously characterized using gas chromatography coupled with flame ionization detection (GC-FID) [35]. This analysis revealed that the extract is rich in saturated fatty acids (51.4 ± 4.89%), followed by polyunsaturated (25.7 ± 3.12%) and monounsaturated fatty acids (19.6 ± 2.88%). The most abundant components include palmitic acid (C16:0, ~40%), arachidonic acid (C20:4, n-6, ~21.1%), and oleic acid (C18:1, n-9, ~14.1%). The omega-3 content was low (~1.2%), whereas the omega-6 content was relatively high. Arachidonic acid, a known precursor of bioactive lipids such as eicosanoids and oxylipins, may contribute to the observed biological activity of Gracilex^®^

### 4.2. Cell Cultures

The human prostate cancer cell lines LNCaP and PC-3 were obtained from the American Type Culture Collection (ATCC, Manassas, VA, USA) and cultured in RPMI-1640 medium (GIBCO, Thermo Fisher Scientific, Waltham, MA, USA), supplemented with 10% heat-inactivated fetal bovine serum (FBS), 100 U/mL penicillin, 100 µg/mL streptomycin, and 1 nM dihydrotestosterone (DHT), as previously described [62,63]. Human umbilical vein endothelial cells (HUVECs) were isolated and cultured according to established protocols [63,64]. Human embryonic kidney (HEK293) and rat pheochromocytoma (PC12) cells were also obtained from ATCC and maintained in DMEM (GIBCO) supplemented with 10% heat-inactivated FBS, 100 U/mL penicillin, and 100 µg/mL streptomycin. All cell cultures were maintained at 37 °C in a humidified incubator with 5% CO_2_.

### 4.3. Microtiter Tetrazolium (MTT) and Cell Counting Assays

The effect of Gracilex^®^ on the viability of LNCaP and PC-3 prostate cancer cell lines was assessed at 24, 48, and 72 h using the MTT assay [3-(4,5-dimethylthiazol-2-yl)-2,5-diphenyltetrazolium bromide]. Briefly, cells were seeded at a density of 5 × 10^5^ cells per well in 24-well plates containing 500 µL of culture medium. After overnight attachment, the medium was replaced with fresh medium containing Gracilex^®^ at the indicated concentrations (1, 10, 50, and 100 µg/mL) or vehicle (0.01% DMSO, control). At each time point, the medium was removed and replaced with 200 µL of HEPES buffer, followed by the addition of 50 µL of MTT solution (2.5 mg/mL in PBS; Sigma-Aldrich, St. Louis, MO, USA). Plates were incubated for 4 h at 37 °C. After incubation, the supernatant was removed, and 200 µL of DMSO, plus 25 µL of Sorensen’s buffer, were added to each well. Absorbance was measured at 570 nm using a microplate reader (EL800, BioTek Instruments, Winooski, VT, USA). Viability data were normalized to vehicle control and expressed as a percentage, allowing comparison across independent experiments.

To further assess proliferation, LNCaP and PC-3 cells were subjected to the trypan blue exclusion assay. Cells were seeded at a density of 5 × 10^2^ cells per well in 24-well plates containing 500 µL of culture medium. After overnight attachment, the medium was replaced with fresh medium containing either Gracilex^®^ (60 µg/mL) or vehicle. Cell growth was monitored over 8–10 days. At each time point, adherent cells were trypsinized and counted using a hemocytometer with the trypan blue exclusion method. In parallel, the effect of Gracilex^®^ on non-malignant cells was evaluated after 48 h of treatment. HUVECs, HEK293, and PC12 cells were treated with the same Gracilex^®^ concentrations, and viability was assessed via the MTT assay as described above.

### 4.4. Immunofluorescence

LNCaP and PC-3 cells were seeded on a glass coverslip (12 mm) into a 24-well plate in fresh medium containing either Gracilex^®^ (60 µg/mL) or vehicle (0.01% DMSO) for 24 h. Cells were then fixed with 4% paraformaldehyde for 30 min at room temperature. Cell permeabilization was performed using 0.1% Triton X-100 in Tris-HCl pH 7.8 for 15 min. Subsequently, the cells were incubated overnight with anti-Ki-67 (1:200 dilution, Abcam, Cambridge, MA, USA) or anti-cleaved caspase-3 (1:100 dilution, Cell Signaling Technologies, Danvers, MA, USA) primary antibody. An Alexa 488–conjugated anti-rabbit IgG (1:500, Molecular Probes, Eugene, OR, USA) and Alexa 594 anti-rabbit IgG conjugated were used as secondary antibodies. Nuclei were visualized using 4′,6-diamidino-2-phenylindole (DAPI) diluted in Tris-HCl pH 7.8 (1:50,000) for 5 min at room temperature. Images were acquired using a Confocal Microscope (LEICA TCS SP8, Buffalo Grove, IL, USA)

### 4.5. Migration and Invasion Assays

Cell migration and invasion capacities were assessed using the CytoSelect™ 96-Well Cell Migration and Invasion Assay Kit (Cell Biolabs, Inc., San Diego, CA, USA), following the manufacturer’s protocol, conducted as previously described [62]. LNCaP and PC-3 cells were cultured in 100 mm plates until reaching 50–60% confluence, then treated with either vehicle (control) or Gracilex^®^ (60 µg/mL) in serum-free RPMI-1640 medium for 24 h.

After treatment, 5 × 10^3^ cells (migration assay) or 2 × 10^3^ cells (invasion assay) were seeded into the upper chamber of each transwell insert containing serum-free RPMI medium with either vehicle or Gracilex^®^ (60 µg/mL). The lower chamber was filled with RPMI supplemented with 10% FBS and either vehicle or Gracilex^®^ to establish a chemotactic gradient. Cells were incubated at 37 °C for 6 h (migration assay) or 24 h (invasion assay). The number of cells that migrated or invaded through the membrane was quantified using a fluorescence-based detection method provided in the kit. Fluorescence was measured using a Synergy2 multi-mode plate reader (BioTek Instruments, Winooski, VT, USA; Ex/Em = 480/520 nm). Data were expressed as the ratio of fluorescence intensity between Gracilex^®^-treated and vehicle-treated cells and reported as mean ± standard deviation (SD) from three independent biological replicates.

### 4.6. Assessment of Systemic Toxicity

To evaluate the systemic toxicity of Gracilex^®^, six-week-old NOD-scid IL2Rgamma^null^ (NSG) mice (Jackson Laboratories, Bar Harbor, ME, USA) were used. Twelve mice were randomly divided into two groups and housed under controlled conditions. The treatment group received oral administration of Gracilex^®^ at a concentration of 300 mg/kg of body weight daily for up to 40 days, while the control group received an equivalent volume of vehicle solution (corn oil). This dosage was calculated based on previously published studies using similar mouse models [35]. Body weights were monitored every three days throughout the experimental period. At the study endpoint, animals were euthanized under anesthesia, and liver tissues were collected for histological analysis. Liver morphology was assessed using hematoxylin and eosin (H&E) staining to detect potential hepatic injury. Additionally, serum triglyceride levels were quantified using a commercial enzymatic assay kit, following the manufacturer’s protocol (Sigma-Aldrich). All animal experimentation was conducted with the approval and under the supervision of the Institutional Animal Care and Use Committee of Pontifical Catholic University of Chile (Protocol number: 221114006).

### 4.7. Xenograft Models

Six-week-old male NOD-scid IL2Rgamma^null^ (NSG) mice (Jackson Laboratories) were randomly assigned to two experimental groups (n = 8 per group) and housed under controlled conditions with ad libitum access to standard chow and water. The groups included (1) a control group receiving vehicle (corn oil) and (2) a treatment group receiving Gracilex^®^ at a dose of 300 mg/kg body weight. Gracilex^®^ was administered via oral gavage three times per week (Monday, Wednesday, and Friday) for five weeks (total of 15 doses). Treatment commenced five days after tumor cell injection to allow sufficient time for initial tumor engraftment, thereby ensuring that any observed effects reflect alterations in the tumor cell proliferative capacity rather than differences in engraftment efficiency. For cell line-derived xenografts, 2 × 10^6^ PC-3 PCa cells were subcutaneously injected into the flanks of the mice. Tumor growth was monitored three times per week over a 5-week period or until tumors reached a maximum volume of 1 cm^3^. Tumor dimensions were measured using calipers, and tumors were excised and weighed at the end of the experiment. At day 32, post-implantation, tumors were surgically collected and processed for histological analysis. All animal procedures were approved by, and conducted under, the supervision of the Institutional Animal Care and Use Committee of the Pontificia Universidad Católica de Chile (Protocol number: 221114006).

### 4.8. Immunohistochemistry

Immunostaining analyses of Ki-67 and cleaved caspase-3 were performed as previously described [65,66]. Antigen retrieval was carried out by incubating tissue sections in 0.01 M sodium citrate buffer (pH 6.0) at 95 °C for 30 min. Endogenous peroxidase activity was quenched using 3% hydrogen peroxide (H_2_O_2_) in methanol. To block nonspecific antibody binding, tissue sections were incubated with 2% bovine serum albumin (BSA) for 20 min at room temperature. Sections were then incubated overnight (12 h) at 4 °C with the following primary antibodies: anti-Ki-67 (1:200, Abcam), anti-Bcl-2 (1:200, Santa Cruz Biotechnology, Dallas, TX, USA), and anti-cleaved caspase-3 (1:100, Cell Signaling). After washing, the sections were incubated for 1 h at room temperature with horseradish peroxidase (HRP)-conjugated secondary antibody (1:100, DAKO, Agilent Technologies, Carpinteria, CA, USA). Signal detection was performed using 3,3′-diaminobenzidine (DAB, DAKO) in the presence of H_2_O_2_, and counterstaining was carried out with Harris hematoxylin. Slides were dehydrated through graded ethanol solutions, cleared in xylene, and mounted with coverslips. Quantitative analysis was performed using ImageJ 1.54p software with the Color Deconvolution plugin. This tool enabled the separation of DAB-specific immunostaining from hematoxylin nuclear staining. For each sample, ten non-overlapping fields were analyzed to calculate the proportion of positively stained cells relative to the total cell nuclei, serving as a normalization parameter for immunoreactivity quantification.

### 4.9. Statistical Analysis

All statistical analyses were performed using GraphPad Prism version 8.0 (GraphPad Software, San Diego, CA, USA). Data are presented as mean ± standard deviation (SD) from at least three independent experiments. Comparisons between two groups were conducted using unpaired Student’s *t*-test, while comparisons involving multiple groups were analyzed using one-way or two-way ANOVA, followed by appropriate post hoc tests when applicable. A *p*-value < 0.05 was considered statistically significant. Linear and nonlinear regression analyses were performed using GraphPad Prism, and only correlations with *r* > 0.9 were considered for interpretation.

## Data Availability

The authors declare that all relevant data supporting the findings of this study are available within the article or from the corresponding author upon request.

## Abbreviations

The following abbreviations are used in this manuscript:

PCa	Prostate Cancer
HUVEC	Human umbilical vein endothelial cells
DHT	Dihydrotestosterone
